# The association between age and adverse events due to biologic disease-modifying antirheumatic drugs in patients with rheumatoid arthritis: A retrospective cohort study

**DOI:** 10.1097/MD.0000000000023861

**Published:** 2020-12-24

**Authors:** Yuzo Ikari, Nobuyuki Yajima, Yusuke Miwa

**Affiliations:** a Division of Rheumatology, Department of Medicine, Showa University School of Medicine, Tokyo; b Department of Healthcare Epidemiology, School of Public Health in the Graduate School of Medicine, Kyoto University, Kyoto; c Center for Innovative Research for Communities and Clinical Excellence, Fukushima Medical University, Fukushima, Japan.

**Keywords:** aged, biologic disease-modifying antirheumatic drugs, drug therapy, drug-related side effects and adverse reactions, rheumatoid arthritis, safety

## Abstract

We examined whether the age of patients with rheumatoid arthritis was associated with adverse events (AEs) caused by biologic disease-modifying antirheumatic drugs (bDMARDs).

Patients with rheumatoid arthritis using bDMARDs from Showa University Hospital, Showa University Northern Yokohama Hospital, and Showa University Koto Toyosu Hospital from January 2005 to December 2017 were eligible for this retrospective cohort study. The maximum observation period was determined to be 1 year. Outcomes in patients older and younger than 75 years were compared. The primary outcome was the rate of drug discontinuation because of AEs caused by bDMARDs. Univariate and multivariate analyses were performed using Pearson's chi-squared test and logistic regression analysis, respectively.

A total of 416 patients were enrolled; median (interquartile range [IQR]): 60.0 (44.3 - 71.0) years and 84.6% women; patients ≥ 75 years were 67/416 (16.1%). The rates of drug discontinuation because of AEs caused by bDMARDs were 10.5% (7/67) in patients 75 years and older and 10.9% (38/349) in those younger than 75 years (relative risk 0.95, 95% confidential interval 0.45-2.24). In logistic regression analysis adjusted for covariates, the rate of drug discontinuation showed no significant difference between the patients ≥ 75 years and the those < 75 years (adjusted odds ratio 0.70, 95% confidential interval 0.29-1.75, *P* = .45).

The rate of drug discontinuation because of AEs caused by bDMARDs was not significantly different between patients 75 years and older and patients younger than 75 years.

## 1 Introduction

In Japan in 2017, elderly people aged 65 years and older accounted for 27.7% of the population. The proportion of elderly individuals will increase in the future, and it is estimated that in 2030, the proportion of elderly individuals will be 31.2%.^[1]^ In Japan, the number of patients with rheumatoid arthritis (RA) is estimated to be 706,000 people, ranging in age from 16 to 74 years (0.6% of the population). Among them, the proportion of individuals aged 60 to 74 years is 61%. Considering that people are living longer with medical advances, the proportion of elderly patients with RA will likely increase in the future.^[2]^

The effectiveness of biologic disease-modifying antirheumatic drugs (bDMARDs) in elderly patients with RA has been demonstrated in several clinical trials.^[3–8]^ However, the usage rate of bDMARDs is low while that of glucocorticoids is high among elderly patients with RA in comparison with that in younger patients with RA.^[9–11]^ It has also been reported that the usage rate of tumor necrosis factor (TNF) inhibitors is low in elderly patients with RA despite their high disease activity.^[10]^ In some clinical studies, age is considered a risk factor for serious infections with the use of bDMARDs.^[12–14]^ In elderly patients with RA, the high risk of infectious adverse events (AEs) caused by bDMARDs may contribute to the low usage rate of bDMARDs despite high disease activity.

In the current literature, clinical studies on elderly patients with RA are limited due to the side effects of drugs and the risk of developing infections. However, a study reported that age was not a risk factor for drug discontinuation due to AEs with TNF inhibitors.^[15]^ Further, it has been reported that TNF inhibitor use does not increase the risk of serious infections in elderly patients with RA compared to non TNF inhibitor users.^[16]^ In this way, there is little evidence to support the association between the age of patients with RA and the rate of AEs onset caused by bDMARDs. Therefore, we aimed to examine whether the age of patients with RA is associated with AEs caused by bDMARDs.

## 2 Methods

### 2.1 Study design

This was a retrospective cohort study.

### 2.2 Patient population

Patients with RA who fulfilled the 2010 ACR/European League Against Rheumatology classification criteria and were using bDMARDs (infliximab, adalimumab, etanercept, golimumab, certolizumab pegol, tocilizumab, abatacept were eligible.^[17]^ For inclusion, we selected all patients with RA who had been older than 15 years when beginning bDMARDs treatment. Participants were recruited from the Showa University Hospital, Showa University Northern Yokohama Hospital and Showa University Koto Toyosu Hospital from January 2005 to December 2017. Sampling was sequential.

### 2.3 Data collection

#### 2.3.1 Variables

All data were collected at the beginning of bDMARDs treatment. The variables were sex, age, Body Mass Index (BMI), disease duration, 28 joint Disease Activity score, erythrocyte sedimentation rate (DAS28 ESR),^[18]^ Health Assessment Questionnaire-Disability Index (HAQ-DI), concomitant prednisolone (PSL) use, PSL dose, concomitant conventional synthetic disease-modifying anti-rheumatic drug (csDMARD) use (methotrexate, bucillamine, salazosulfapyridine, tacrolimus, leflunomide, mizoribine, and iguratimod), and the presence of interstitial lung disease (ILD), diabetes mellitus (DM) and chronic kidney disease (CKD). Disease duration was the period from the day of diagnosis of RA to the day of starting bDMARDs. DAS28 ESR provided a global summative and continuous score for disease activity assessment, and it has been widely used in clinical trials and practice. HAQ-DI is a self-report questionnaire that measures function including the performance of activities of daily living. ILD, DM, and CKD were diagnosed by the attending doctor.

#### 2.3.2 Exposure

We classified patients with RA into 2 groups: elderly and non-elderly. Age was defined as the age at which time the biologic was begun. Elderly patients with RA were defined as those 75 years and older. The reason for this is because the Japan Geriatric Society defines elderly people as those 75 years and older.^[19]^ In many countries including Japan, elderly people are defined as being 65 years and older. However, people between the ages of 65 and 74, which have traditionally been regarded as elderly, often maintain their physical and psychological well-being, and those who are capable of active social activities are mostly occupied.

#### 2.3.3 Outcomes

The primary outcome was drug discontinuation because of AEs caused by bDMARDs. The maximum observation period was determined to be 1 year. Temporary discontinuation of bDMARDs was not considered as the termination of bDMARDs. Temporary discontinuation was defined as restarting the same biologic within 1 year of drug interruption. AEs were defined as all adverse events leading to the discontinuation of bDMARDs excluding primary and secondary invalid, remission and patient hope. If an event had no obvious causal relationship with bDMARDs, the AE was not counted, for example, an elective operation for joint replacement. The judgment of AEs caused by bDMARDs was performed by 2 rheumatologists. If the opinions of the 2 rheumatologists differed, they decided to collect by discussion. The secondary outcome was drug discontinuation because of infectious AEs caused by bDMARDs. Age was defined as the age at which time the biologic was begun. So, if the patients reached the age of 75 during the analysis period, they could not belong to both age groups. Furthermore, outcomes were counted only once per participant. After the outcome occurred, the follow was discontinued.

### 2.4 Statistical analysis

Sex, csDMARDs use and the presence of ILD, DM, and CKD were analyzed as categorical variables; DAS28 ESR and HAQ-DI were analyzed as numerical variables; and age, disease duration, and the glucocorticoid dose were analyzed as continuous variables. Summary statistics are presented as IQR and as a percentage (%). Univariate analysis was performed using Pearson's chi-squared test. Logistic regression analysis was performed to assess the association between the rate of drug discontinuation because of AEs caused by bDMARDs. Confounding factors that we selected were sex, glucocorticoid dose, csDMARDs, and the presence of ILD, DM, and CKD. These covariates were chosen based on past literature and clinical importance.^[20–23]^ Missing variables were replaced by multiple imputations. Furthermore, we performed sensitivity analyses that changed the definition of elderly individuals from 75 years and older to 65 years and older. A 2-sided *P*-value < .05 was considered statistically significant. All statistical analyses were conducted using STATA 14 version 14.2 (StataCorp LP, College Station, TX).

## 3 Results

### 3.1 Baseline characteristics

The characteristics of patients are shown in Table 1. Four hundred sixteen patients were included in the analysis. Patients’ age was median [IQR]: 60.0 [44.3 - 71.0] years, and 84.6% were women. Patients aged 75 years and older accounted for 67/416 patients (16.1%). The minimum age of the patients is 17 years old, and the maximum age of the patients is 90 years old. Compared to the group younger than 75 years, the group 75 years and older had more severe disease activity (DAS28 ESR, median [IQR]: 5.2 [4.4 – 6.3] versus [vs] 4.8 [3.5 – 5.8], *P* = .008), severe physical dysfunction (HAQ-DI, median [IQR]: 1.13 [0.38 – 1.63] vs 0.38 [0.13 – 1.0], *P* < .001). For administered bDMARDs, the group 75 years and older had high usage rate of abatacept (23.9% vs 11.2%, *P* = .005) and a lower usage rate of TNF inhibitor (52.2% vs 71.6%, *P* = .002). The bDMARDs were administered for the first biologic in 85.6%, for the second biologic 10.0%, and for third or latter biologic 4.4%, respectively. The group 75 years and older had more patients treated with PSL (68.2% vs 49.6%, *P* = .006) and less patients treated with methotrexate (55.2% vs 77.9%, *P* < .001). Regarding comorbidities, patients 75 years and older had a higher prevalence of ILD (31.3% vs. 14.0%, *P* < .001) and CKD (46.3% vs 10.9%, *P* < .001) than those younger than 75 years. There was no significant difference between the groups for the prevalence of DM (22.4% vs 13.5%, *P* = .06). During the observation period, 250/416 patients (60.1%) continued bDMARDs. There were 129/416 patients (31.0%) confirmed discontinuation of bDMARDs. Within the observation period, 37/416 patients (8.9%) became unfollowable. The reasons for discontinuation are shown in Table 2. The most common reason for discontinuation was due to AEs caused by bDMARDs (45/416 [10.8%]). Discontinuation of primary invalid was 8.4% (35/416) and secondary invalid was 7.2% (30/416).

**Table 1:** Baseline characteristic of patients.

	Total n = 416	Patients aged ≥ 75 years n = 67	Patients aged <75 years n = 349	*P*-value
Age (years), median (IQR)	60.0 (44.3–71.0)	78 (76–81)	55 (42–66.5)	<.001
Data missing	0	0	0	
Female sex, n (%)	352 (84.6)	52 (77.6)	300 (86.0)	.08
Data missing	0	0	0	
BMI (IQR)	21.1 (19.0–23.4)	21.1 (19.1–23.8)	21.0 (19.0–23.1)	.24
Data missing	4	0	4	
Disease duration (days), median (IQR)	1397 (457–4045)	1672 (491–5274)	1308 (443–3932)	.27
Data missing	6	1	5	
DAS28 ESR, median (IQR)	4.9 (3.8–5.9)	5.2 (4.4–6.3)	4.8 (3.5–5.8)	.008
Data missing	4	1	3	
HAQ-DI, median (IQR)	0.5 (0.25–1.13)	1.13 (0.38–1.63)	0.38 (0.13–1.0)	<.001
Data missing	110	24	86	
TNF inhibitor, n (%)	285	35 (52.2)	250 (71.6)	.002
TCZ, n (%)	76	16 (23.9)	60 (17.2)	.19
ABT, n (%)	55	16 (23.9)	39 (11.2)	.005
Data missing	0	0	0	
1^st^ biologic, n (%)	351 (85.6)	58 (89.2)	293 (84.9)	
2^nd^ biologic, n (%)	41 (10.0)	6 (9.2)	35 (10.1)	
≥ 3^rd^ biologic, n (%)	18 (4.4)	1 (1.5)	17 (4.9)	
Data missing	6	2	4	
PSL, n (%)	216 (52.6)	45 (68.2)	171 (49.6)	.006
Data missing	5	1	4	
PSL dose (mg), median (IQR)	2 (0–5)	3 (0–5)	0 (0–5)	.10
Data missing	5	1	4	
MTX, n (%)	309 (74.3)	37 (55.2)	272 (77.9)	<.001
Data missing	0	0	0	
csDMARDs, n (%)	371 (89.2)	56 (83.6)	315 (90.3)	.10
Data missing	0	0	0	
ILD, n (%)	70 (16.8)	21 (31.3)	49 (14.0)	<.001
Data missing	0	0	0	
DM, n (%)	62 (14.9)	15 (22.4)	47 (13.5)	.06
Data missing	0	0	0	
CKD, n (%)	69 (16.9)	31 (46.3)	38 (10.9)	<.001
Data missing	0	0	0	
Observation period day, median (IQR)	365 (196–365)	358 (187–365)	365 (197–365)	.40
Observation period until discontinuation by AEs day, median (IQR)	113 (51–209)	63 (32–134)	113 (60–134)	.06
Data missing	0	0	0	

**Table 2:** Reasons for discontinuation of biologics.

	Total n = 129	Patients aged ≥75 years n = 22	Patients aged <75 yr n = 107
Primary invalid	35	4	31
Secondary invalid	30	9	21
AEs (Infectious AEs)	45 (12)	7 (3)	38 (9)
Remission	8	0	8
Patient hope	8	1	7
Other	3	1	2

### 3.2 Drug discontinuation because of AEs

The rates of drug discontinuation because of AEs caused by bDMARDs were 10.5% (7/67) in patients 75 years and older and 10.9% (38/349) in those younger than 75 years (relative risk 0.95, 95% confidential interval [CI] 0.45-2.24, *P* = .92). AEs due to bDMARDs that led to drug discontinuation are shown in Table 3. AEs excluding infectious AEs include rash, cytopenia, infusion reaction and elevation liver enzymes and so on. In the logistic regression analysis adjusting for sex, the glucocorticoid dose, csDMARDs, and the presence of ILD, DM, and CKD, the rate of drug discontinuation because of AEs caused by bDMARDs showed no significant difference between the elderly and non-elderly groups (adjusted odds ratio 0.70, 95% CI 0.29-1.75, *P* = .45) (Table 4). Regarding the secondary outcome, there was no significant difference in the rate of drug discontinuation because of infectious AEs caused by bDMARDs between the 2 groups in the logistic regression analysis (adjusted odds ratio 1.39, 95% CI 0.32-5.96, *P* = .66) (Table 5).

**Table 3:** AEs due to biologics that led to drug discontinuation.

	Total n = 45	Patients aged ≥75 years n = 7	Patients aged <75 yr n = 38
Bacterial pneumonia	7	1	6
Herpes zoster	3	1	2
PCP	2	1	1
Rash	9	2	7
Cytopenia	1	0	1
Exacerbation of ILD	1	0	1
Infusion reaction	3	0	3
Allergy	1	0	1
Anaphylactic reaction	2	0	2
Elevation liver enzymes	3	0	3
Dyspnea	1	0	1
Malignant lymphoma	1	0	1
cervical cancer	2	0	2
liver cancer	1	1	0
eosinophilia	1	1	0
Paf	1	0	1
gum bleeding	1	0	1
pulmonary embolism	1	0	1
stomach adenoma	1	0	1
fever	1	0	1
lymphatic edema	1	0	1
lupus-like syndrome	1	0	1

**Table 4:** Association between the age of patients and drug discontinuation by all cause AEs (Elderly definition: ≥75 yr).

	Number, (%)	Unadjusted Odds ratio [95% CI]	Adjusted Odds ratio [95% CI]	*P*-value
Age, years <75 yr (n = 349)	38 (10.9)	reference	reference	–
Age, years ≥75 yr (n = 67)	7 (10.5)	0.95 [0.34–2.3]	0.70 [0.29–1.75]	.45

**Table 5:** Association between the age of patients and drug discontinuation by infectious AEs.

	Number, (%)	Unadjusted Odds ratio [95% CI]	Adjusted Odds ratio [95% CI]	p-value
Age, years < 75 years (n = 349)	9 (2.6)	reference	reference	–
Age, years ≥75 years (n = 67)	3 (4.5)	1.77 [0.30–7.34]	1.39 [0.32–5.96]	0.66

### 3.3 Sensitivity analysis

In the sensitivity analysis, the rate of drug discontinuation because of AEs due to bDMARDs was not significantly different between the group 65 years and older and the group younger than 65 years in the logistic regression analysis (adjusted odds ratio 0.99, 95% CI 0.49-2.02, *P* = .99) (Table 6).

**Table 6:** Sensitivity analysis of the association between the age of patients with RA and drug discontinuation by all cause AEs (Elderly definition: ≥65 years).

	Number, (%)	Unadjusted Odds ratio [95% CI]	Adjusted Odds ratio [95% CI]	*P*-value
Age, yr <65 yr (n = 253)	25 (9.9)	reference	reference	–
Age, years ≥65 yr (n = 163)	20 (12.3)	1.27 [0.65–2.49]	0.99 [0.49–2.02]	.99

## 4 Discussion

In this study, we examined whether the age of patients with RA was associated with AEs caused by bDMARDs. In the logistic regression analysis adjusted for co-variables (sex, the glucocorticoid dose, csDMARDs, and the presence of ILD, DM and CKD), the rate of drug discontinuation because of AEs caused by bDMARDs showed no significant difference between the elderly and non-elderly groups. The same result was obtained in the sensitivity analysis using 65 years as the cutoff value for elderly. Concerning the secondary outcome, there was no significant difference in the rate of drug discontinuation because of infectious AEs due to bDMARDs between the elderly and non-elderly groups.

Our study showed that the rate of drug discontinuation because of AEs caused by bDMARDs was not significantly different between the elderly and non-elderly groups. This is similar to results of previous studies.^[16,24,25]^ In some clinical studies, age is considered a risk factor for serious infections with the use of bDMARDs.^[20,26,27]^ Three mechanisms are assumed to increase the rate of AEs. The first is a reduction in immunity due to aging. Because of age-related acquisition of the immune system, one's declining immunologic function against infection becomes a risk for onset of infectious AEs. RA itself also accelerates immunosenescence.^[28]^ The second is polypharmacy. With increasing age, there is a tendency toward polypharmacy.^[29,30]^ Intake of many drugs concomitantly increase the risk of AEs due to drug interactions and complicate disease management.^[31]^ The third is the number of comorbidities. The number of comorbidities increases in elderly patients with RA. However, our data did not show an increased incidence of AEs in elderly patients. Kawashima et al. showed that there is no significant difference in the incidence of serious infections in RA patients who are 65 years and older between those treated with bDMARDs and non-bDMARDs.^[24]^ In addition, low-dose glucocorticoid use (prednisolone 1-4 mg/d) has been shown to be a risk factor of serious infections in elderly RA patients treated with bDMARDs.^[24]^ Yves-Marie Pers et al. demonstrated that the rate of discontinuation due to AEs of tocilizumab use was not significantly different between patients over 65 and under 65 years.^[25]^ In another study, although increasing age was an independent risk factor for serious infection in patients on anti-TNF therapy, there was no difference in relative risk of infection in patients on anti-TNF therapy in the older population.^[16]^ Conversely, a previous study has shown that the rate of drug discontinuation because of AEs caused by bDMARDs in RA patients 65 years and older is correlated with the age at start bDMARDs. However, this is an exploratory study and age is thought to contribute slightly (hazard ratio 1.05, 95% CI 1.00-1.10, p = 0.024).^[26]^ In addition, the rate of drug discontinuation because of AEs caused by bDMARDs in RA patients is reported to be higher in the elderly, but in this study, more confounding factors were included in the multivariate analysis compared to the number of results.^[20]^ Another study found that the incidence of abatacept AEs in RA patients was associated with an increase in age, but multivariate analysis was not performed. Accordingly the relationship between age and the incidence of abatacept AEs in RA patients is not clear.^[27]^ From the above, there are inconsistent results that the incidence of AEs is higher in elderly RA patients.

This study has some limitations. First, it was a research study performed in a university hospital; so, there is no external validity of the patients. The background of patients with RA may be different between a university hospital and a family practice clinic. In general, patients at university hospitals are more complicated and refractory. There is a possibility that our results do not apply to all elderly patients with RA. Second, this study included data from January 2005 to December 2017. This is a long term in terms of changes in the use of bDMARDs in RA. A better understanding of the safety of bDMARDs and the availability of new bDMARDs may bias our results. Third, our study may have unmeasured covariates, and we consider disease duration as 1 of them. Disease duration has been reported as a risk factor for drug discontinuation because of AEs caused by bDMARDs. However, in a past study, drug discontinuation was minimally higher because of longer disease duration (hazard ratio 1.02, 95% CI 1.00-1.04, *P* = .027).^[15]^ Therefore, it may not have a significant impact on our results. Furthermore, we did not investigate a comorbidity index such as modified Rheumatic Disease Comorbidity Index.^[32]^ In our dataset, we have not investigated research items about modified Rheumatic Disease Comorbidity Index. Fourth, we could not check for minor AEs that would not result in the discontinuation of bDMARDs. This study only extracted AEs that led to discontinuation. Drug continuation is considered an important parameter for evaluating drug efficacy and AEs.^[33]^ AEs leading to drug discontinuation were considered clinically important AEs. Further, our study included patients who discontinued bDMARDs for reasons other than AEs. This may not be able to pick up patients who discontinue bDMARDs in the future due to AEs.

Our study has 2 strengths. First, to our knowledge, this is the first study to adjust for important co-variables related to discontinuation due to AEs caused by bDMARDs. The results shown here were obtained by including co-variables that had been indicated in previous studies. Second, in our study, treatment choice and criteria of biologic discontinuation were determined by the judgment of the attending doctor and patient hope. These decisions are based on actual clinical practice. Based on our study's results, if the introduction of bDMARDs is believed to be the best RA treatment option for an elderly patient with RA, efforts are needed to minimize AEs caused by bDMARDs. These efforts include infection prevention, drug adjustment to reduce polypharmacy, and prevention of comorbidities, such as DM.

## 5 Conclusions

Our results demonstrated that the rate of drug discontinuation because of AEs caused by bDMARDs was not significantly different between patients who were 75 years and older and those younger than 75 years.

## 6 Compliance with ethical standards

Personal information linked to the research participants was anonymized. We obtained written informed consent from all of the patients who enrolled in the study. Participants who could not provide informed consent were excluded from the study. The study received approval from the Bio-Ethics Committee of the Department of Medicine, Showa University School of Medicine (No. 1435).

## Author contributions

All authors are responsible for the integrity of the study and the final manuscript.

**Conceptualization:** Yuzo Ikari, Nobuyuki Yajima.

**Data curation:** Yuzo Ikari, Nobuyuki Yajima, Yusuke Miwa.

**Formal analysis:** Yuzo Ikari, Nobuyuki Yajima.

**Funding acquisition:** Nobuyuki Yajima, Yusuke Miwa.

**Investigation:** Yuzo Ikari.

**Methodology:** Yuzo Ikari, Nobuyuki Yajima.

**Project administration:** Yuzo Ikari, Nobuyuki Yajima.

**Resources:** Yuzo Ikari, Nobuyuki Yajima, Yusuke Miwa.

**Supervision:** Nobuyuki Yajima, Yusuke Miwa.

**Validation:** Yuzo Ikari, Nobuyuki Yajima, Yusuke Miwa.

**Visualization:** Yuzo Ikari.

**Writing – original:** Yuzo Ikari.

**Writing – review & editing:** Nobuyuki Yajima, Yusuke Miwa.
